# Paraneoplastic cerebellar degeneration with anti-Yo antibodies and an associated submandibular gland tumor: a case report

**DOI:** 10.1186/s12883-022-02684-4

**Published:** 2022-05-02

**Authors:** Takeshi Imai, Kensuke Shinohara, Kenji Uchino, Hirohisa Okuma, Futaba Maki, Kiyoshi Hiruma, Yasushi Ariizumi, Yoshihisa Yamano

**Affiliations:** 1 Department of Neurology, Kawasaki Municipal Tama Hospital: Kawasaki Shiritsu Tama Byoin, 1-30-37, Shukugawara Tama-ku Kawasaki-shi, Kawasaki, Kanagawa Japan; 2 Department of Neurology, Shin Yurigaoka General Hospital: Shinyurigaoka Sogo Byoin, Kawasaki, Kanagawa Japan; 3 Department of Otorhinolaryngology, Kawasaki Municipal Tama Hospital: Kawasaki Shiritsu Tama Byoin, Kawasaki, Kanagawa Japan; 4 Department of Diagnostic Pathology, St Marianna University School of Medicine Yokohama Seibu Hospital: Sei Marianna Ika Daigaku Yokohama-shi Seibu Byoin, Yokohama, Kanagawa Japan; 5 Department of Internal Medicine, Division of Neurology, St Marianna University School of Medicine: Sei Marianna Ika Daigaku, Kawasaki, Kanagawa Japan

**Keywords:** Paraneoplastic cerebellar degeneration, Submandibular gland tumor, Anti-Yo antibodies, Salivary duct carcinoma, Paraneoplastic syndrome

## Abstract

**Background:**

As a debilitating syndrome, paraneoplastic cerebellar degeneration (PCD) remains challenging to treat. Further, anti-Yo antibody (directed against human cerebellar degeneration-related protein 2) detection in patients with PCD is associated with unsatisfactory responses to existing therapies. Here, we present the case of a 60-year-old woman who developed PCD with anti-Yo antibodies and a submandibular gland tumor.

**Case presentation:**

A 60-year-old woman presented with a 5-day history of unsteadiness of gait and inadequate coordination of her extremities, along with truncal instability. Although walking without aid was possible, dysmetria of all four limbs, trunk, and gait ataxia was observed. While routine biochemical and hematological examinations were normal, the patient’s blood was positive for anti-Yo antibodies. When the neurological symptoms deteriorated despite administration of intravenous methylprednisolone, fluorodeoxyglucose-positron emission tomography (FDG-PET) and computed tomography (CT) images with contrast enhancement were performed, which showed a tumor in the left submaxillary gland. She underwent total left submandibular gland resection, including the tumor; histological and immunohistochemical results revealed a salivary duct carcinoma. She was administered intravenous methylprednisolone, followed by 10 plasma exchange sessions, intravenous immunoglobulins, and cyclophosphamide therapy. Following treatment, her symptoms were not alleviated, even after the reduction of anti-Yo titers.

**Conclusions:**

Although tumor detection was delayed, early tumor detection, diagnosis, and PCD treatment are essential because any delay can result in the progression of the disorder and irreversible neurological damage. Therefore, we recommend that the possibility of a salivary gland tumor should be considered, and whole-body dual-modality CT, including the head and neck, and FDG-PET should be performed at the earliest for patients with well-characterized paraneoplastic antibodies when conventional imaging fails to identify a tumor.

## Background

Paraneoplastic cerebellar degeneration (PCD), a devastating paraneoplastic syndrome (PNS) characterized by subacute cerebellar ataxia, dysarthria, and ocular dysmetria, is a collection of neurological disorders resulting from tumor-induced autoimmunity against cerebellar antigens [1, 2]. Highly specific anti-neuronal antibodies in the serum and the cerebrospinal fluid (CSF) represent key diagnostic biomarkers of PCD. Approximately 30 different antibodies are associated with this condition [3]. Some anti-neuronal antibodies, such as the anti-Yo antibodies that are directed against human cerebellar degeneration-related protein 2, are only associated with PCD [4]. Anti-Yo–mediated PCD tends to occur predominantly in women aged approximately 60 years and is mainly associated with gynecologic malignancy (the ovary, uterus, and breast) [5]. While PCD remains a difficult condition to treat, anti-Yo PCD is associated with some of the poorest response rates to standard therapies [2].

In this report, we present the case of a patient with PCD, anti-Yo antibodies, and an associated submandibular gland tumor. To the best of our knowledge, this is the first reported case of PCD associated with a submandibular gland tumor.

## Case presentation

A 60-year-old woman with no relevant past medical history, including alcohol or family history, was admitted to our department because of progressive gait instability. Two weeks before admission, the patient reported difficulties in writing. On admission, she presented with a 5-day history of unsteadiness of gait and inadequate coordination of her extremities, along with truncal instability.

The findings on admission were as follows: height, 154 cm; body weight, 50 kg; body temperature, 36.2 °C; blood pressure, 125/85 mmHg; and peripheral capillary oxygen saturation, 98% (room air). On examination, her cognition was normal, spontaneous nystagmus was absent, and smooth-pursuit eye movements were normal. Manual muscle testing was performed bilaterally, and a score of 5/5 was noted for the upper and lower extremities. Although walking without aid was possible, she showed dysmetria of all four limbs, trunk, and gait ataxia.

Routine biochemistry and hematological test results were normal. Magnetic resonance imaging (MRI) of the head did not show any signal change in the brain parenchyma. In particular, no abnormalities were observed in the brainstem or cerebellum. The CSF was clear, with a cell count of 30 cells/μL (76% mononuclear cells, 24% polymorphonuclear cells), glucose concentration of 84 mg/dL (124 mg/dL blood glucose), protein concentration of 82 mg/dL, and positive oligoclonal bands. No evidence of malignant cells was seen on CSF cytology. Consecutive serum and CSF analyses did not indicate active infections (herpes simplex virus, varicella-zoster virus, Epstein–Barr virus, and cytomegalovirus) and systemic autoimmune causes. Further biochemical examination revealed normal levels of vitamins B1 and B12.

We suspected PNS and tested for neuronal autoantibodies. The patient tested strongly positive for the anti-Yo antibody in the blood but negative for other antineuronal antibodies (Hu, Ri, Tr, CV2, amphiphysin, recoverin, SOX1, titin, zic4, and GAD65). All neuronal autoantibodies were confirmed on immunoblotting with recombinant proteins.

With the PCD diagnosis, intravenous methylprednisolone (1000 mg/day) was administered for 3 days, followed by seven sessions of plasma exchange. However, the patient’s neurological symptoms deteriorated, with progressive staggering vertigo, nausea, nystagmus in all directions of gaze, dysarthria, dysphagia, and especially severe truncal ataxia, disabling her walk.

At the same time, thoracic and abdominal computed tomography (CT) and an enhanced abdominal MRI to identify the primary tumor were unremarkable.

**Fig. 1:**
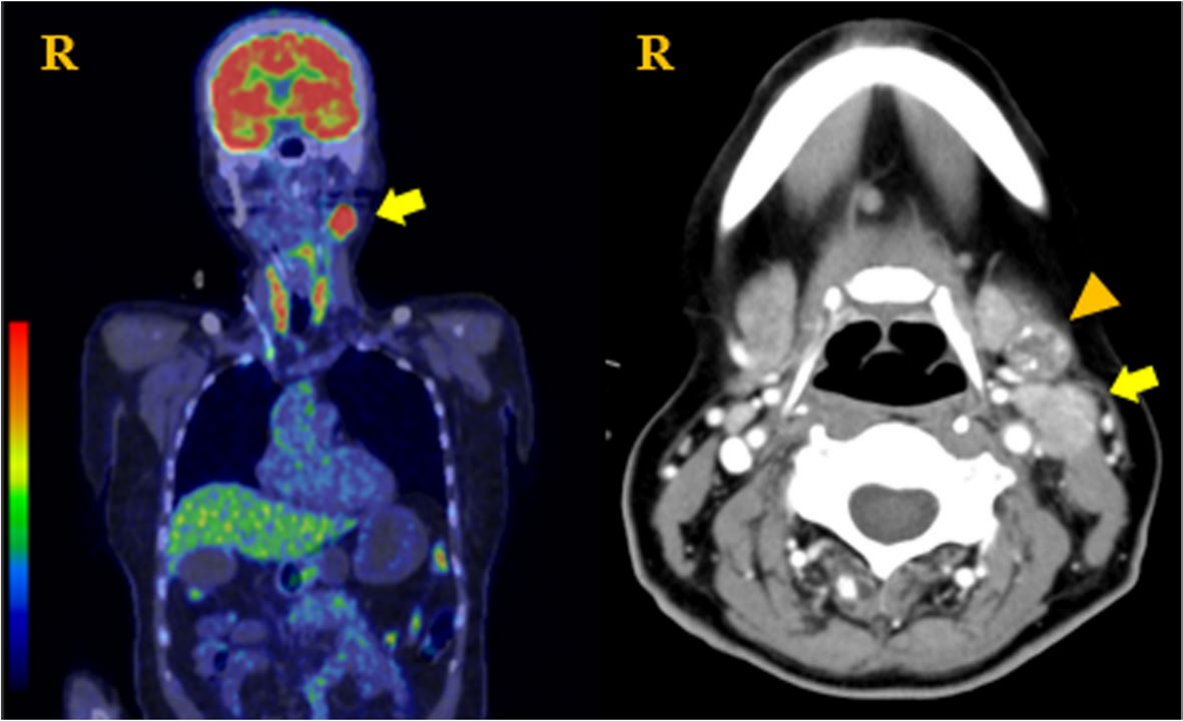
FDG-PET (fluorodeoxyglucose-positron emission tomography) and computed tomography (CT) images with contrast enhancement. FDG-PET showed abnormal hot spots in the left neck (arrow). CT images with contrast enhancement showed a tumor associated with the left submaxillary gland, showing contrast effects along the periphery (arrow) and an increase in lymph nodes with calcification (arrowhead)

Next, we performed fluorodeoxyglucose-positron emission tomography (FDG-PET) at another hospital to identify the primary tumor because we could not perform it in our hospital. The FDG-PET showed abnormal hot spots in the left neck. CT with contrast enhancement showed a tumor associated with the left submandibular gland, showing contrast effects along the periphery and an increase in lymph nodes with calcification (Fig. 1).

**Fig. 2:**
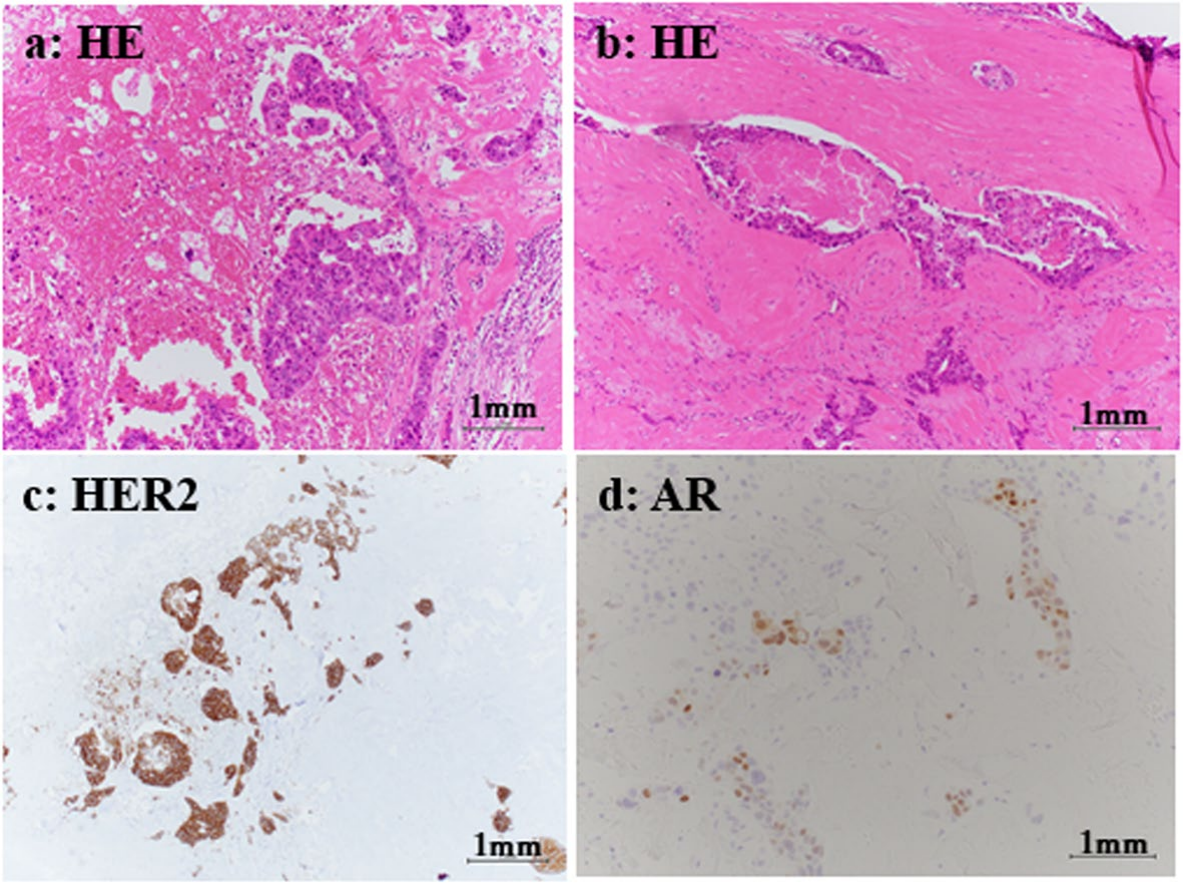
Histopathology of the left submandibular gland tumor. The tumor cells had large round nuclei in tubular and cribriform structure (**a**) with comedo necrosis (**b**). Immunohistochemistry revealed that these cells were positive for human epidermal growth factor receptor 2 (**c**) and focally positive for androgen receptor (**d**)

After otorhinolaryngology consultation, the patient underwent total left submandibular gland resection, including the tumor, and modified neck dissection for the ipsilateral metastatic lymph nodes. Histopathology showed tumor cells with large round nuclei and tubular and cribriform structures with comedo necrosis. Immunohistochemistry revealed that these cells were positive for human epidermal growth factor receptor 2 (HER2) and focally positive for androgen receptor (AR) (Fig. 2). Histological and immunohistochemical analyses revealed salivary duct carcinoma (SDC). We diagnosed PCD with anti-Yo antibodies and an associated SDC with reference to the diagnostic criteria [6].

Tumor resection was initiated, which halted the disease progression and improved the vertigo, nausea, and nystagmus. The patient received intravenous methylprednisolone 1000 mg/day for 3 consecutive days, followed by 10 sessions of plasma exchange during the following months. Intravenous immunoglobulin (IVIG) was administered for 5 additional days, and cyclophosphamide therapy was performed. However, this treatment did not alleviate the symptoms in all four limbs, trunk, and gait ataxia, even though the anti-Yo titers reduced following treatment. The patient was transferred to another hospital 4 months after admission because there was no improvement in her neurological symptoms; she is still in the hospital and left bedridden.

## Discussion and conclusions

PCD is a collection of neurological disorders resulting from tumor-induced autoimmunity against cerebellar antigens. Ataxic syndrome associated with anti-Yo antibodies is the most common variant of PCD.

As a pancerebellar syndrome, ataxia affects both the trunk and limbs, but the onset can be asymmetric in a subset of patients [2, 7]. Symptoms suggestive of brainstem involvement, such as dysarthria, nystagmus, diplopia, and dysphagia, are often noted [2, 7] and appear to reach a plateau within 6 months of onset, even without any intervention [7, 8]. Cognitive and psychiatric morbidity, especially memory loss and emotional lability, is also common in these patients. However, concomitant dysarthria can make this syndrome difficult to diagnose [8]. Although a case with extensive MRI signal abnormality in the cerebellar hemispheres early during anti-Yo PCD has been reported [9], brain MRI in the anti-Yo syndrome can be normal, and cerebellar atrophy is usually only seen after the disease is well established [7]. 18-FDG PET can show reductions in mean metabolic rate in the cerebellum [9]. Furthermore, diffuse Purkinje cell degeneration with CD8 lymphocytic infiltration and microglial activation has been found at autopsy in a patient with a normal MRI scan [10], indicating that the correlation between imaging findings and pathology is not clear.

Patients with anti-Yo antibodies have a median survival of 13 months, and anti-Yo PCD is associated with some of the most unsatisfactory response rates to conventional therapies [2]. The syndrome progresses over weeks to months, with most patients being in wheelchairs or bedridden during this time [7]. Our patient had progressive vertigo, disequilibrium, vertical nystagmus, and diplopia, which gradually worsened over 1 month. Her symptoms exacerbated during this period to the point where the patient became completely bedridden. Although the prognosis for anti-Yo PCD is almost uniformly poor, some reports indicate that young patients and those with low initial disability appear likely to show a good response to treatment [11, 12].

Some reports indicate benefits from early antitumor therapies like surgery and chemotherapy [13]. Initiation of immunosuppression therapy within the first month is thought to be important in impacting the clinical course of paraneoplastic neurological syndromes [12, 14]. Prior case reports of IVIG used in anti-Yo PCD found that treatment initiation within a month of the onset of symptoms was associated with a good outcome [15]. PCD treatment includes immunoglobulins, corticosteroids, and chemotherapy in association with supportive therapy.

PCD, a rare but devastating PNS, is most commonly associated with breast and gynecological cancers. It has also been associated with Hodgkin lymphoma; small cell lung, gastric, esophageal, prostate, and bladder cancers; and melanoma. Given the association of PNS with breast and gynecological cancers, a majority of the patients are females [2, 7]. On the other hand, reports of its association with head and neck cancer are rare. Although PCD cases with anti-Hu antibodies associated with parotid gland tumors have been reported [16], this is the first reported case of PCD associated with a submandibular gland tumor.

Although our patient had typical clinical features and was positive for anti-Yo antibodies, the primary tumor was unknown even after performing chest and abdominal CT with contrast enhancement, abdominal ultrasonography, and MRI.

It has been reported that an FDG-PET or CT is a highly accurate diagnostic modality for detecting underlying primary tumors in PNS-suspected patients [17, 18]. In cases where the initial tumor screen is negative, patients should be followed up at regular intervals with scans [15].

SDC is a rare tumor with an estimated incidence of 1–3% among all malignant salivary gland tumors. It is often observed in men and occurs more commonly in those aged > 50 years [19, 20]. Although there were no symptoms directly caused by the ductal carcinoma in our case, pain and facial palsy are commonly observed as subjective symptoms [21, 22]. SDC is a high-grade malignant salivary gland tumor associated with poor prognosis, frequent recurrence, and metastasis. It is histologically similar to invasive mammary ductal carcinoma and is characterized by cribriform and central necrosis known as “comedo necrosis” [23]. In addition, immunostaining frequently provides positive results for HER2 and AR [24, 25].

In the present case, tumor detection was delayed. Thus, early tumor detection, PCD diagnosis, and treatment are crucial as a late diagnosis may result in tumor progression and irreversible neurological damage. When standard imaging fails to detect a possible salivary gland tumor, whole-body dual-modality CT, including the head and neck, and FDG-PET should be performed immediately for patients with well-characterized paraneoplastic antibodies.

## Data Availability

Because of privacy concerns, we are unable to provide any additional clinical data due to the unique characteristics of this case.

## Abbreviations

FDG-PET: Fluorodeoxyglucose-positron emission tomography
CT: Computed tomography
SDC: Salivary duct carcinoma
HER2: Human epidermal growth factor receptor 2
AR: Androgen receptor
PNS: Paraneoplastic syndrome
PCD: Paraneoplastic cerebellar degeneration
MRI: Magnetic resonance imaging
IVIG: Intravenous immunoglobulin
CSF: Cerebrospinal fluid
